# Adaptive evolution and functional significance of the *PPARGC1A* gene across diverse animal species

**DOI:** 10.1002/ece3.11238

**Published:** 2024-10-03

**Authors:** Seyed Mahdi Hosseini, Farman Ullah, Muhammad Zulfiqar Ahmad, Majid Pasandideh, Aixin Liang, Guohua Hua, Liguo Yang

**Affiliations:** ^1^ National Center for International Research on Animal Genetics, Breeding and Reproduction (NCIRAGBR) College of Animal Science and Technology, Huazhong Agricultural University Wuhan China; ^2^ Department of Plant Breeding and Genetics, Faculty of Agriculture Gomal University Dera Ismail Khan Pakistan; ^3^ Department of Animal Science, Faculty of Agriculture Shahrekord University Shahrekord Iran; ^4^ Hubei Hongshan Laboratory Huazhong Agricultural University Wuhan China

**Keywords:** adaptive evolution, maximum likelihood, positive selection, PPARGC1A gene

## Abstract

Codon‐based analyses of the *PPARGC1A* gene across 38 vertebrate species were deployed to elucidate patterns of evolutionary change. Employing maximum likelihood assessments through MEGA, we scrutinized 447 codon positions addressing the entire coding region, excluding positions mired by gaps or missing data. Distinct codons manifested variance in selection pressures, particularly codons 4, 11, 66, and 123, which exhibited positive dN‐dS values suggestive of positive selection. Codon 137 displayed the most pronounced dN‐dS value, signifying intensified selective advantage. Meanwhile, codons 30 and 90 portrayed near‐neutral scores, indicative of purifying selection. Complementary computational methods (IFEL, REL, FUBAR, and SLAC) confirmed positive selection at specific codon sites, with varying degrees of corroboration. The integration of mixed‐effect modeling (MEME) identified episodic diversifying selection, pinpointing codons that underwent selection episodes in certain lineages. Refined codon model selection lent insight into substitution rates, revealing nuanced degrees of evolutionary conservation among different codons. Supporting these genetic insights, the phylogenetic analysis highlighted relationships among the *PPARGC1A* sequences and domain analysis confirmed conserved features across species, while protein–protein interaction networks suggested a complex web of functional interdependencies. These findings advance our understanding of the *PPARGC1A* gene's evolutionary trajectory and underscore the gene's potential adaptive significance within diverse vertebrate lineages.

## Graphical abstract

This study conducts codon‐based analyses of the *PPARGC1A* gene across 38 vertebrate species, revealing distinct patterns of evolutionary change. Positive selection pressures were observed at specific codon sites, particularly 4, 11, 66, and 123, with codon 137 displaying the most pronounced selective advantage. Computational methods confirmed these findings, and mixed‐effect modeling identified episodic diversifying selection. The research provides nuanced insights into substitution rates, evolutionary conservation, phylogenetic relationships, and functional interdependencies within the *PPARGC1A* gene, advancing our understanding of its evolutionary trajectory and adaptive significance across diverse vertebrate lineages.

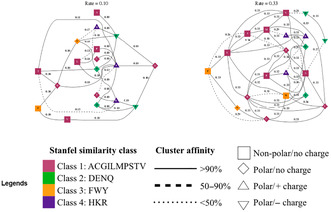

## 1 INTRODUCTION

Evolutionary biology helps us understand gene function, genetic modification procedures, and gene transfer, including the study of point mutations, gene and genomic replication, probability, heritability, and extensive genomic relationships (Manolio et al., 2009). Molecular evolution focuses on genes associated with beneficial traits and their propagation through the population via selective breeding (Sabeti et al., 2002). Numerous evaluative research studies have been conducted on different genes (De Matos et al., 2013; Farmanullah et al., 2020; Gaur et al., 2017; Huang & Liao, 2015; Neves et al., 2016; Wang et al., 2023). For example, Ahmad et al. (2017) examined molecular evolutionary signatures that may influence selection processes in the *MC1R* gene in goats and identified evolutionary traces that may affect adaptation to different environments. Another study maximized the search for positive selection signatures involving mammalian *ILS 1–37* using a variety of approaches. The results propose hypotheses related to IL functions, which should be further investigated using mutagenesis and crystallographic approaches (Neves et al., 2014). Studying the evidence for positive selection in the *PPARGC1A* gene helps us better understand the molecular underpinnings of adaptation and the critical role of metabolism in species' survival. It provides an exciting interface between evolutionary biology, ecology, and medicine to study how life evolves and maintains itself in a dynamic world (Gregory, 2009).

The *PPARGC1A* gene, also known as *PGC‐1α* (peroxisome proliferator‐activated receptor gamma coactivator 1‐alpha), is a transcriptional coactivator that plays a key role in regulating cellular energy metabolism. It is crucial to control genes involved in energy production through processes like gluconeogenesis, fatty acid oxidation, and mitochondrial biogenesis (Hosseini et al., 2021; Liang & Ward, 2006). In the context of vertebrate evolution, examining the *PPARGC1A* gene can give insights into how different species have adapted their metabolic processes to various environmental challenges and demands. The *PPARGC1A* gene is highly conserved across different species, suggesting that it plays a fundamental and vital role in energy metabolism that has been preserved throughout evolution. Conservation implies that the gene is subjected to purifying selection, where changes are typically not favored because they may disrupt essential functions (LeMoine et al., 2010).

However, there are instances of adaptive evolution within the *PPARGC1A* gene when looking across different vertebrate species. Different species might show unique adaptations in the *PPARGC1A* protein that suggest positive selection at play—mutations that provided selective advantages may have been conserved. For instance, species living in cold climates might have undergone changes in *PPARGC1A* that enhance their ability to generate heat through non‐shivering thermogenesis in brown adipose tissue (Popov et al., 2017). An interesting aspect of the evolutionary analysis could also involve the gene's role in disease resistance. For instance, certain variants of the *PPARGC1A* protein may contribute to better handling of metabolic diseases, which can be a factor in the longevity and survival of some species (Cheng et al., 2018). To study *PPARGC1A*'s molecular evolution, scientists use a variety of techniques such as phylogenetic analysis, comparative genomics, and tests for adaptive selection (e.g., dN/dS ratio comparison) (Ahmad et al., 2018; Farmanullah et al., 2020). These analyses can illuminate the selection pressures acting on the gene and show where in the gene's sequence changes are more likely to occur.

In summary, the evolutionary analysis of the *PPARGC1A* gene involves studying its conservation and variation among species, understanding how these changes correlate with species' adaptations to their environment, and observing its impact on species survival and fitness. Such studies are essential to grasp the complex interplay between genetics and evolution in shaping the physiological capabilities of organisms. We hypothesize that the *PPARGC1A* gene has undergone positive selection in animal species that have adapted to extreme environmental conditions, resulting in significant molecular variations that correlate with enhanced metabolic efficiency and survival advantage compared to species without such adaptations.

## 2 MATERIALS AND METHODS

### 2.1 Animal selection and gene sequence data retrieval

The gene sequence data critical for this study was obtained from the National Center for Biotechnology Information (NCBI) Gene database. To encompass a broad evolutionary representation, we included *PPARGC1A* gene sequences from 38 different vertebrate species, covering a diverse phylogenetic range. These species were specifically selected to represent not only a comprehensive cross‐section of vertebrate classes but also to capture a variety of ecological niches and diverse metabolic requirements.

For each of the chosen species, we accessed the gene sequences related to the PPARGC1A gene and documented the corresponding NCBI accession numbers. These unique identifiers are provided in Table 1 of the study and juxtaposed with the species names for quick reference. Accumulating these accession numbers allows the study's replication and lends credibility to ongoing or future research endeavors, guaranteeing traceability and reproducibility of the findings. Table 1 presented in the study serves as a directory that lists the species along with three types of accession numbers. By documenting these accession numbers, we have created a systematic and organized approach to gene sequence data retrieval, thus ensuring that each piece of genetic information can be accurately and reliably sourced for the study.

**TABLE 1:** List of species and accession number of the NCBI gene bank database used in the study.

S. no.	Species	Accession no.	mRNA accession No.	Protein accession No.
1	Human	NC_000004.12	NM_001354826.2	NP_001317681.1
2	House Mouse	NC_000071.6	XM_030254205.1	XP_017176206.1
3	Norway rat	NC_005113.4	XM_008770219.1	XP_008768441.1
4	Chimpanzee	NC_036883.1	XM_016951412.2	XP_016806900.1
5	White‐tufted‐ear marmoset	NC_013898.1	XM_017970644.1	XP_017826132.1
6	Cattle	NC_037333.1	XM_015471552.2	XP_024848828.1
7	Painted turtle	NC_024223.1	XM_005303448.3	XP_005303505.1
8	Sheep	NC_040257.1	XM_004009738.4	XP_004009787.1
9	Rhesus monkey	NC_041758.1	XM_015138111.2	XP_014993597.1
10	Damara mole‐rat	NW_022900947.1	XM_010610445.2	XP_010608746.1
11	Chinese tree shrew	NW_006159931.1	XM_027774375.1	XP_027630176.1
12	Water buffalo	NC_037551.1	XM_025290240.1	XP_006080562.1
13	Domestic ferret	NW_004569232.1	XM_004762682.2	XP_004762742.1
14	Chinese hamster	NW_003614424.1	XM_003507779.4	XP_035315299.1
15	Miniopterusnatalensis	NW_015504714.1	XM_016218251.1	XP_016073739.1
16	Egyptian rosette	NW_015494598.1	XM_016154824.1	XP_016010317.1
17	Sooty mangabey	NW_012005712.1	XM_012045316.1	XP_011900708.1
18	Chinese soft‐shelled turtle	NW_005871016.1	XM_025179018.1	XP_006138221.1
19	Cheetah	NW_020834726.1	XM_027047192.1	XP_026902993.1
20	Domestic cat	NC_018726.3	XM_019829685.2	XP_023109020.1
21	Giant panda	NC_048228.1	XM_034670962.1	XP_034526853.1
22	Gray short‐tailed opossum	NC_008805.1	XM_007496657.2	XP_007496718.1
23	Long‐tailed chinchilla	NW_004955480.1	XM_005402463.2	XP_005402521.1
24	Naked mole‐rat	NW_004624755.1	XM_021260219.1	XP_012928538.1
25	Northern white‐cheeked gibbon	NC_044400.1	XM_030800951.1	XP_030656810.1
26	Przewalski horse	NW_007673183.1	XM_008513563.1	XP_008511785.1
27	Prairie vole	NW_004949103.1	XM_026788617.1	XP_005366076.1
28	Pacific walrus	NW_004450440.1	XM_004402853.2	XP_004402912.1
29	Pig‐tailed macaque	NW_012011067.1	XM_011751473.2	XP_011749769.1
30	Sumatran orangutan	NC_036907.1	XM_009239844.2	XP_024101288.1
31	Wild Bactrian camel	NC_045697.1	XM_032494896.1	XP_032350787.1
32	Western European hedgehog	NW_006804142.1	XM_007523875.2	XP_007523939.1
33	Weddell seal	NW_006385223.1	XM_006746098.2	XP_006746161.1
34	American beaver	NW_017869532.1	XM_020161454.1	XP_020017051.1
35	Australian saltwater crocodile	NW_017728948.1	XM_019553218.1	XP_019408762.1
36	Koala	NW_018343954.1	XM_020992475.1	XP_020848146.1
37	Olive baboon	NC_044978.1	XM_031664085.1	XP_031519947.1
38	Zebrafish	NC_007118.7	XM_017357140.2	XP_017212627.1

*Note*: Gene accession no.: This number provides the specific location of the DNA sequences within the NCBI GenBank sequence database. mRNA accession no.: This number gives access to the curated mRNA sequences in the NCBI RefSeq database, associated with the *PPARGC1A* gene for the respective species. Protein accession no.: This number details the protein products corresponding to the *PPARGC1A* gene as recorded in the NCBI RefSeq database.

### 2.2 Protein–protein interaction (PPI) network analysis

Network analysis of protein–protein communications is also crucial for further knowing the molecular function of the *PPARGC1A* gene. Gene interactions with the *PPARGC1A* gene were divined utilizing STRING‐specific correlation analysis (version 9.1, http://www.string‐db.org/) (Franceschini et al., 2012; Li et al., 2017). Web Server Database Biological communications were used to recognize and detect protein communications. The standard cutoff value was used as the sum of 0:4. Highly bound and essential biological function proteins were shown in the intermediate nodes. Identification, documentation, and evaluation were based on the number of connections between each node's proteins and the degree of similarity.

### 2.3 Domain analysis

The domain architecture of the *PPARGC1A* gene across various species was analyzed using TBTool version 1.068 (Chen et al., 2020). Domain structures were predicted to verify the presence and conservation of the respective domains within *PPARGC1A* sequences.

### 2.4 Phylogenetic analysis

Molecular Evolutionary Genetics Analysis version 7 (MEGA 7) software was employed to construct a phylogenetic tree via the maximum likelihood (ML) method (Kumar et al., 2016). The reliability of the tree was assessed by bootstrapping with 1000 replicates, and branches corresponding to partitions reproduced in less than 50% of bootstrap replicates were collapsed. Different colors were assigned to visualize distinct phylogenetic groupings.

### 2.5 Selection pressure analysis

Selection pressures on *PPARGC1A* gene sequences were estimated using the mechanistic‐empirical combination (MEC) model provided by the SELECTION web server tool version 2.2 (http://selecton.tau.ac.il/) that can visualize and estimate the selection pressures on genes (Stern et al., 2007). It uses color coding to show positive, neutral, and purifying selection on the sequence alignment or phylogenetic tree. Results were visualized with color‐coded highlights to represent different types of selection: yellow and brown for positive selection, gray and white for neutral selection, and purple for negative selection.

### 2.6 Maximum likelihood analysis of natural selection

For each codon, this study presents estimates of the number of synonymous (s) and nonsynonymous (n) substitutions, along with the number of sites estimated to be synonymous (S) and nonsynonymous (N). These estimates were produced using joint maximum likelihood reconstructions of ancestral states under a Muse‐Gaut codon substitution model (Muse & Gaut, 1994) and a Tamura‐Nei nucleotide substitution model (Tamura & Nei, 1993). A tree topology was computed automatically to estimate maximum likelihood (ML) values. The test statistic dN‐dS detects codons that may have undergone positive selection. Here, dS represents the number of synonymous substitutions per site (s/S), and dN represents the number of nonsynonymous substitutions per site (n/N). A positive test statistic suggests an excess of nonsynonymous substitutions. In such cases, the probability of rejecting the null hypothesis, which posits neutral evolution (*p*‐value), is calculated (Pond & Frost, 2005; Suzuki & Gojobori, 1999). A *p*‐value of less than .05 is considered statistically significant at the 5% level and is thus highlighted. Normalized dN‐dS values for the test statistic are obtained using the total number of substitutions in the tree, measured in expected substitutions per site, facilitating comparisons across datasets. Maximum likelihood computations of dN and dS were conducted using the HyPhy (Hypothesis testing using Phylogenies) software package (Pond et al., 2005).

### 2.7 Evolution of the mixed‐effect model

Evolution of the mixed‐effect model (MEME) is a combination of fixed effects to identify instances of diverse episodic selection and pervasive positive selection at the individual branch site level and Fast Unconstrained Bayesian Approximation (FUBAR) using the Markov Chain Monte Carlo (MCMC) that ensures robustness. Against the incorrect feature of the model at predefined sites through the Bayesian approximation approach (Murrell et al., 2012). The *p*‐values are derived using a mixture of Chi‐squared distributions, and the *q*‐values are obtained through Simes' procedure, which controls the false discovery rate under a strictly neutral null hypothesis (and is likely to be conservative) (Farmanullah et al., 2020).

### 2.8 Evolutionary rate analysis

Evolutionary rate clustering was examined using structured genetic algorithm models from the DATAMONKEY web server (www.datamonkey.org). Each cluster was labeled with the maximum likelihood estimate of its inferred rate, and the nodes represented the biochemical properties and Stanfel class annotations of the residues.

### 2.9 Ramachandran plot prediction

The conformational ethos of the *PPARGC1A* protein structure, including the amino acid and atom proximity, was taken into account using Ramachandran plots predicted through VADAR (http://vadar.wishartlab.com/). VADAR (Volume, Area, Dihedral Angle Reporter) is a compilation of more than 15 different algorithms and programs for analyzing and assessing peptide and protein structures from their PDB coordinate data. VADAR produces extensive tables and high‐quality graphs for quantitatively and qualitatively assessing protein structures determined by X‐ray crystallography, NMR spectroscopy, 3D‐threading, or homology modeling (Willard et al., 2003).

### 2.10 Codon‐based measures of molecular evolution

Analyzing molecular evolution through codon‐based measures involves bioinformatics techniques, often utilizing software and computational tools to scrutinize mutations in codons across DNA or RNA sequences. MEGA is employed to assess the cumulative impact of three types of codon alterations: synonymous, nonsynonymous, and ambiguous. The findings are then visualized using color plots (Kumar et al., 2016).

### 2.11 Conserved region analysis

Using motif analysis, the conservation of specific regions within the *PPARGC1A* gene from different species was ascertained and visualized concerning phylogenetic positioning.

Through the Clustal Omega method (Sievers & Higgins, 2018), we could align sequences from different species, identify motifs (short recurring patterns that are presumed to have a biological function), and create visual representations of these conserved elements about the evolutionary tree of the species being studied.

## 3 RESULTS

### 3.1 Maximum likelihood analysis of natural selection codon‐by‐codon

The analysis involved 38 nucleotide sequences with codon positions including first, second, third, and noncoding regions. All positions with gaps and missing data were excluded, resulting in a total of 447 positions in the final dataset. Evolutionary analyses were conducted using MEGA. Table 2 displays data from a maximum likelihood analysis looking for evidence of positive selection within the *PPARGC1A* gene, on a codon‐by‐codon basis. Codons 4, 11, 66, and 123 show a dN‐dS value of 0.50. Codon 137 exhibits the highest dN‐dS value (1.96), which substantially exceeds the other codons. Codons 30 and 90 show low dN‐dS values close to zero (0.00 and 0.08, respectively), which could imply that these sites are under purifying selection or neutral evolution. Codons 54, 61, 101, and 132 have relatively high dN values compared with dS values, indicated by dN‐dS of 0.99, 0.82, 0.96, and 0.96, respectively.

**TABLE 2:** Codon‐wise positive selection analysis of PPARGC1A gene.

Codon	Codon start	Triplet	Syn (s)	Nonsyn (n)	Syn sites (S)	Nonsyn sites (N)	dS	dN	dN‐dS	*p*‐Value
4	493	GGC	0.00	1.00	1.00	2.00	0.00	0.50	0.50	.67
11	514	GCA	0.00	1.00	1.00	2.00	0.00	0.50	0.50	.67
30	697	CAA	0.50	1.50	0.65	1.93	0.77	0.78	0.00	.75
32	706	AAG	0.50	1.50	0.82	2.00	0.61	0.75	0.14	.71
54	772	ACC	1.00	4.00	0.99	2.01	1.01	1.99	0.99	.47
61	793	AAC	0.00	2.00	0.56	2.44	0.00	0.82	0.82	.66
62	889	AGC	1.00	4.00	0.65	2.33	1.53	1.72	0.19	.70
64	895	AGA	1.33	3.67	0.89	1.96	1.50	1.87	0.37	.61
66	901	AAA	0.50	2.50	0.66	2.18	0.76	1.14	0.38	.66
70	925	AAA	0.00	1.00	0.56	2.24	0.00	0.45	0.45	.80
72	931	AAG	0.00	1.00	0.74	2.07	0.00	0.48	0.48	.74
73	934	TCC	2.00	4.00	1.00	1.98	2.00	2.02	0.02	.68
83	988	CAA	0.00	1.00	0.70	1.74	0.00	0.58	0.58	.71
90	1009	TCT	1.00	2.00	1.00	1.84	1.00	1.08	0.08	.72
101	1042	CCC	0.00	2.00	0.92	2.08	0.00	0.96	0.96	.48
119	1096	CTC	0.00	1.00	0.97	2.03	0.00	0.49	0.49	.68
120	1099	TCT	0.00	1.00	0.90	2.03	0.00	0.49	0.49	.69
123	1108	GCA	0.00	1.00	1.00	2.00	0.00	0.50	0.50	.67
132	1318	AAG	0.00	2.00	0.73	2.09	0.00	0.96	0.96	.55
137	1339	ACT	2.75	10.25	0.88	2.02	3.11	5.06	1.96	.35
142	1387	TTT	0.00	1.00	0.53	2.44	0.00	0.41	0.41	.82
146	1399	GAC	0.50	2.50	0.57	2.43	0.87	1.03	0.16	.72

*Note*: Codon: Number identifying the specific codon in the gene sequence that is under investigation. Codon start: The nucleotide position in the gene sequence at which the codon starts. Triplet: The nucleotide triplet that makes up the codon. Syn (s): The observed number of synonymous substitutions, which are changes in the DNA sequence that do not result in a change in the amino acid sequence. Nonsyn (n): The observed number of nonsynonymous substitutions, which are changes in the DNA sequence that result in a change in the amino acid sequence. Syn sites (S): Estimated number of sites in the codon that could potentially undergo synonymous changes without altering the amino acid. Nonsyn sites (N): Estimated number of sites in the codon that could potentially undergo nonsynonymous changes, altering the amino acid. dS: The calculated rate of synonymous substitutions per synonymous site. dN: The calculated rate of nonsynonymous substitutions per nonsynonymous site. dN‐dS: The difference between dN and dS rates; it is used to infer the type of natural selection acting on a codon. Positive values can indicate positive selective pressure, whereas negative values indicate purifying selection, n and values around zero suggest neutral evolution.

The data in this table suggests varying degrees of selective pressure across the different codons analyzed within the *PPARGC1A* gene. Some codons appear to be under stronger positive selection than others.

### 3.2 Codon model selection

Table 3 displays the results of codon model selection for the *PPARGC1A* gene across different organisms, evaluated using the modified Bayesian information criterion (mBIC). Three classes of models, with varying numbers of rate classes (N), have been assessed to determine the rates of synonymous (dS) and nonsynonymous (dN) substitutions in the gene.

**TABLE 3:** Codon model selection based on modified Bayesian information criterion (mBIC) of *PPARGC1A* gene from different organisms.

Classes	Models	Credible	mBIC	ΔmBIC	dN/dS (rates in class)		
1	1	0	40,844.8		0.22/75		
2	3227	813	40,562.5	282.28	0.10/30	0.33/45	
3	1060	14	40,562.8	−0.27	0.09/30	0.32/41	0.65/4.0

*Note*: *N*: number of rate classes included in models; Models: genetic algorithm models; Credible: all the models evaluated by genetic algorithm within 9.21 mBIC unit (the best model has credible values 0.01 or >1); mBIC: modified Bayesian information criterion; ΔmBIC: mBIC for *N* rate classes compared to *N* − 1 rate classes; dN/dS: maximum likelihood estimates for each rate class.

Class 1 Model is the simplest model with a single rate class. It has the highest mBIC value (40844.8), which generally indicates a less optimal model compared to others with lower mBIC values. It shows a dN/dS ratio of 0.22/75, suggesting low levels of positive selection since the rate of nonsynonymous substitutions is low in comparison with synonymous substitutions. Class 2 model is a more complex model with 3227 models evaluated. It has a substantially lower mBIC value (40,562.5), improving upon the Class 1 Model by 282.28 points. This indicates a better fit. The rate classes here are split into two groups, with dN/dS ratios of 0.10/30 and 0.33/45, respectively. This suggests different levels of selection pressure on different sets of codons within the gene. Class 3 Model is similar to Class 2 but with only 1060 models evaluated and an even lower mBIC value (40,562.8). The negative ΔmBIC value represents a statistical anomaly possibly due to the rounding errors or model complexity. This class posits three rate classes with dN/dS ratios of 0.09/30, 0.32/41, and 0.65/4.0, suggesting varying selective pressure across different codon sites, with the last ratio indicating a stronger positive selection in a small subset of codons. The mBIC values in Table 3 serve to compare models, with lower values indicating a better fit to the observed data. The ΔmBIC shows the improvement of each model over the model with one fewer rate class. Furthermore, the “Credible” column indicates the models that fall within 9.21 mBIC units of the best model, which would be deemed credible. A credible value of 1 or more, or close to 0.01, shows high confidence in model selection.

Overall, the best‐fitted model according to the mBIC criterion includes two rates, as indicated by the significant improvement over the basic single rate model both in terms of likelihood (151.63 log(L)) and in mBIC (282.28 points). The genetic algorithm used here tested 4288 models to find the best fit, demonstrating an extensive search for the optimal model representing the evolutionary pressures on the *PPARGC1A* gene across different organisms.

### 3.3 Best codon model selection

Table 4 presents the best codon model selection for the *PPARGC1A* gene, based on the modified Bayesian information criterion (mBIC), and it focuses on specifics regarding rate classes and the types of amino acid changes that occur.

**TABLE 4:** Best codon model selection based on modified Bayesian information criterion (mBIC) of *PPARGC1A* genes.

dN/dS	Rates in class	Mean model averaged dN/dS	Stanfel class changes	Polarity changes	Charge changes
0.10	30	0.11	26	10	23
0.33	45	0.33	20	22	8

*Note*: dN/dS (nonsynonymous/synonymous substitution rates): This ratio is used to infer the type of natural selection acting on a protein‐coding gene. A ratio less than 1 suggests purifying selection, equal to 1 suggests neutral evolution and greater than 1 suggests positive selection. Rates in class: The number of different rate classes in the model. A rate class corresponds to a subset of codons that are evolving at the same rate, and different rate classes indicate heterogeneity in the evolutionary rates across codon sites. Mean model averaged dN/dS: An average of the dN/dS ratios across the different evaluated models, providing an overall estimate that considers the different rate classes. This gives a more nuanced understanding of the selective pressures by averaging across models. Stanfel class changes: The number of times a substitution leads to changes in structural features, such as the stability of the protein, due to changes in the amino acid's size or composition. Polarity changes: The number of substitutions that result in changes between polar and non‐polar amino acids, which can affect the protein's interaction with water or other molecules. Charge changes: The number of substitutions that lead to changes in the amino acid charge, potentially affecting the protein's structure, function, and interactions due to electrostatic charges.

The first line indicates a dN/dS ratio of 0.10 across 30 rate classes, which shows strong purifying selection (favors evolutionary conservation as the dN/dS is far below 1). The mean averaged dN/dS ratio is 0.11, closely aligning with the class‐specific ratio. This class has a high number of Stanfel class changes (26) and charge changes (23), which could indicate that although there is strong conservation when changes do happen, they have significant structural and functional impacts. There are fewer polarity changes (10), suggesting that changes affecting solubility or interactions with water are less frequent. The second line reflects a dN/dS ratio of 0.33 across 45 rate classes, still suggesting purifying selection but less stringent than in the first class. The mean model‐averaged dN/dS is equal to the class‐specific ratio at 0.33. This group has fewer Stanfel class changes (20) but a higher number of polarity changes (22), indicating a greater evolutionary openness to changes that affect the amino acid's behavior concerning water or its polarity. There are fewer charge changes (8), suggesting that alterations affecting charge are less common or less accepted in these codons.

Overall, Table 4 appears to describe a gene that is mostly conserved (as indicated by dN/dS ratios less than 1), yet it also shows evidence of some evolutionary flexibility, given the presence of class changes—including in Stanfel class, polarity, and charge—suggesting that some variability is tolerated or even selected for through evolution.

### 3.4 Evolutionary evidence of positive selection

Table 5 presents the results of four different computational methods used to identify sites in the *PPARGC1A* gene that are under positive selection. Positive selection at a particular site in a gene suggests that mutations at that site may confer some selective advantage and are being favored by natural selection.

**TABLE 5:** Sites under positive selection within the *PPARGC1A* genes, employing various approaches.

IFEL	REL	FUBAR	SLAC
Positive sites	Positive codon	Positive sites	Positive sites
(*p*‐value)	(Bayes Factor)	(Posterior probability)	(*p*‐value)
31 (.06), 37 (.02), 40 (.08), 52 (.05), 154 (.09), 394 (.08), 521 (.02), 525 (.03), 636 (.09)	18 (78), 20 (521), 21 (905), 22 (1523), 23 (328), 25 (160), 26 (1237), 27 (226), 28 (1978), 29 (59,269), 33 (3968), 34 (790), 35 (4774), 39 (688), 42 (43,622), 45 (72), 46 (72), 47 (10,482), 48 (15488), 49 (3), 51 (1), 52 (5)	23 (.88), 28 (.81), 29 (.90), 31 (.81), 33 (.94), 37 (.84), 46 (.96), 49 (.87), 51 (.99), 52 (.95), 394 (.98), 644 (.98)	51 (.073), 394 (.031), 644 (.028)

*Note*: IFEL (Internal fixed effects likelihood): Analyzes individual codon sites to test if any have a significant rate of nonsynonymous changes, indicating positive selection. Sites are identified by *p*‐values, with lower values indicating stronger evidence for positive selection. REL (Random effects likelihood): Estimates nonsynonymous and synonymous rate changes at each site to identify those under selection. It uses a Bayes Factor to support the presence of positive selection, with higher values suggesting stronger evidence. FUBAR (Fast, Unconstrained Bayesian AppRoximation): Uses a Bayesian approach to identify sites under selection, providing posterior probabilities that a certain rate class is more likely. Values closer to 1 indicate a stronger belief in positive selection at that site. SLAC (Single Likelihood Ancestor Counting): Compares the expected to the observed number of synonymous and nonsynonymous substitutions. Positive selection sites are identified with *p*‐values, and as with IFEL, lower values indicate stronger evidence.

Our results indicate that IFEL identified eight sites under positive selection based on their *p*‐values. REL identified a larger number of codon sites with Bayes factors suggesting strong evidence for positive selection. FUBAR identified 12 sites with high posterior probabilities indicating a strong belief in positive selection. SLAC identified three sites under positive selection with low *p*‐values. Site 52 is identified by both IFEL and FUBAR methods, and site 394 is identified by IFEL, FUBAR, and SLAC. Site 29 stands out in the REL analysis with an extremely high Bayes Factor, which is supported by a high posterior probability in the FUBAR method but is not identified by the *p*‐value‐based methods (IFEL and SLAC), suggesting some discrepancy between methods that could warrant further investigation.

Generally speaking, some discrepancies among the methods are normal due to differences in statistical approaches and underlying assumptions. Reconciliation of results from multiple approaches lends credence to the idea that certain sites are under positive selection and can provide a more comprehensive view of the evolutionary processes acting on the *PPARGC1A* gene.

### 3.5 Mixed‐effect evolution analysis

Table 6 is the result of using the mixed effects model of evolution (MEME) to detect episodic diversifying selection in the *PPARGC1A* gene. MEME allows for the detection of positive selection that affects only a few branches of the phylogenetic tree, hence the term “episodic” (Murrell et al., 2012). The table lists various codons in the *PPARGC1A* gene along with the reported rates of synonymous and nonsynonymous substitutions. For example, at codon 33, the synonymous rate (*α*) and the nonsynonymous rate under purifying selection (*β*−) are both 0.64 with a high probability (Pr[*β* = *β*−] = .91). However, there is a low probability (.09) of a much higher nonsynonymous rate under positive selection (*β*+ = 920.26), as indicated by Pr[*β* = *β*+]. The *p*‐value of .00 strongly suggests that episodic positive selection has happened here, and the *q*‐value of 0.01 confirms the reliability of this result when considering multiple tests. Conversely, at codon 394, while there is a high probability (Pr[*β* = *β*+] = 1.00, i.e., 100%) for a certain level of positive selection (*β*+ = 1.61), the *p*‐value and *q*‐value are both relatively higher than some other codons (though still relatively small at 0.01 and 0.28 respectively), potentially indicating a less robust case for positive selection or a more conservative estimate.

**TABLE 6:** Mixed‐effect model evolution (MEME) based episodic diversifying selection of *PPARGC1A* genes.

Codon	Α	*β*−	Pr[*β* = *β*−]	*β*+	Pr[*β* = *β*+]	*p*‐Value	*q*‐Value
33	0.64	0.64	.91	920.26	.09	.00	0.01
37	0.00	0.00	.79	32.61	.21	.00	0.00
40	0.00	0.00	.84	12.34	.16	.00	0.04
44	1.14	0.00	.84	2693.79	.16	.00	0.04
45	0.73	0.00	.87	2694.02	.13	.00	0.00
46	0.47	0.00	.83	136.52	.17	.00	0.00
48	0.00	0.00	.77	83.32	.23	.00	0.00
49	0.41	0.24	.69	175.13	.31	.00	0.00
51	1.46	0.22	.72	160.32	.28	.00	0.00
52	0.38	0.00	.73	2693.98	.27	.00	0.00
372	0.33	0.00	.98	80.60	.02	.01	0.30
394	0.00	0.00	.00	1.61	1.00	.01	0.28
467	0.00	0.00	.98	44.54	.02	.00	0.04
556	0.00	0.00	.94	219.63	.06	.01	0.28
644	0.00	0.00	.00	1.56	1.00	.01	0.28
747	1.30	0.13	.98	618.08	.02	.01	0.28
751	3.12	0.00	.98	165.51	.02	.01	0.29
791	1.68	0.00	.98	172.67	.02	.00	0.17
793	0.39	0.00	.98	151.96	.02	.00	0.04
850	0.00	0.00	.98	27.34	.02	.00	0.03
857	0.00	0.00	.98	28.65	.02	.01	0.29
861	0.00	0.00	.97	16.32	.03	.00	0.07
870	0.00	0.00	.98	53.30	.02	.00	0.11
871	0.00	0.00	.98	72.38	.02	.00	0.16
883	0.00	0.00	.98	61.26	.02	.00	0.12
901	0.35	0.00	.90	17.43	.10	.00	0.04
911	0.00	0.00	.98	23.15	.02	.00	0.12
913	0.45	0.00	.98	87.22	.02	.00	0.10
935	0.45	0.00	.96	71.28	.04	.00	0.11
938	0.61	0.00	.94	22.38	.06	.00	0.18
944	0.00	0.00	.98	143.85	.02	.00	0.06

*Note*: Codon: The position of the amino acid in the protein sequence coded by the gene. *α* (alpha): The synonymous substitution rate, which is the rate at which mutations that do not change the amino acid occur. These are generally assumed to be selectively neutral. *β*− (beta minus) and Pr[*β* = *β*−]: The estimated rate of nonsynonymous substitutions that do not lead to a change in function (purifying selection) and the probability that the substitution rate is purifying, respectively. *β*+ (beta plus)and Pr[*β* = *β*+]: The estimated rate of nonsynonymous substitutions that lead to a change in function (positive selection) and the probability that the substitution rate is under positive selection, respectively. *p*‐value: The probability that the observed data would occur under the null hypothesis (which usually posits no positive selection). A low *p*‐value suggests that the null hypothesis can be rejected, usually taken as evidence for positive selection. *q*‐value: The smallest false discovery rate at which the test may be called significant. It is a measure used to control for multiple testing. A lower *q*‐value indicates greater reliability in the result.

Generally, codons with both low *p*‐values and *q*‐values would be considered the strongest candidates for episodic diversifying selection. As MEME tests episodic selection, the presence of one or a few instances of positive selection (*β*+) across the tree (at given codons) is enough to be picked up by this method, even if most of the time, the evolutionary pressure at that site is neutral or purifying.

### 3.6 Evolutionary rate clustering

**FIGURE 1:**
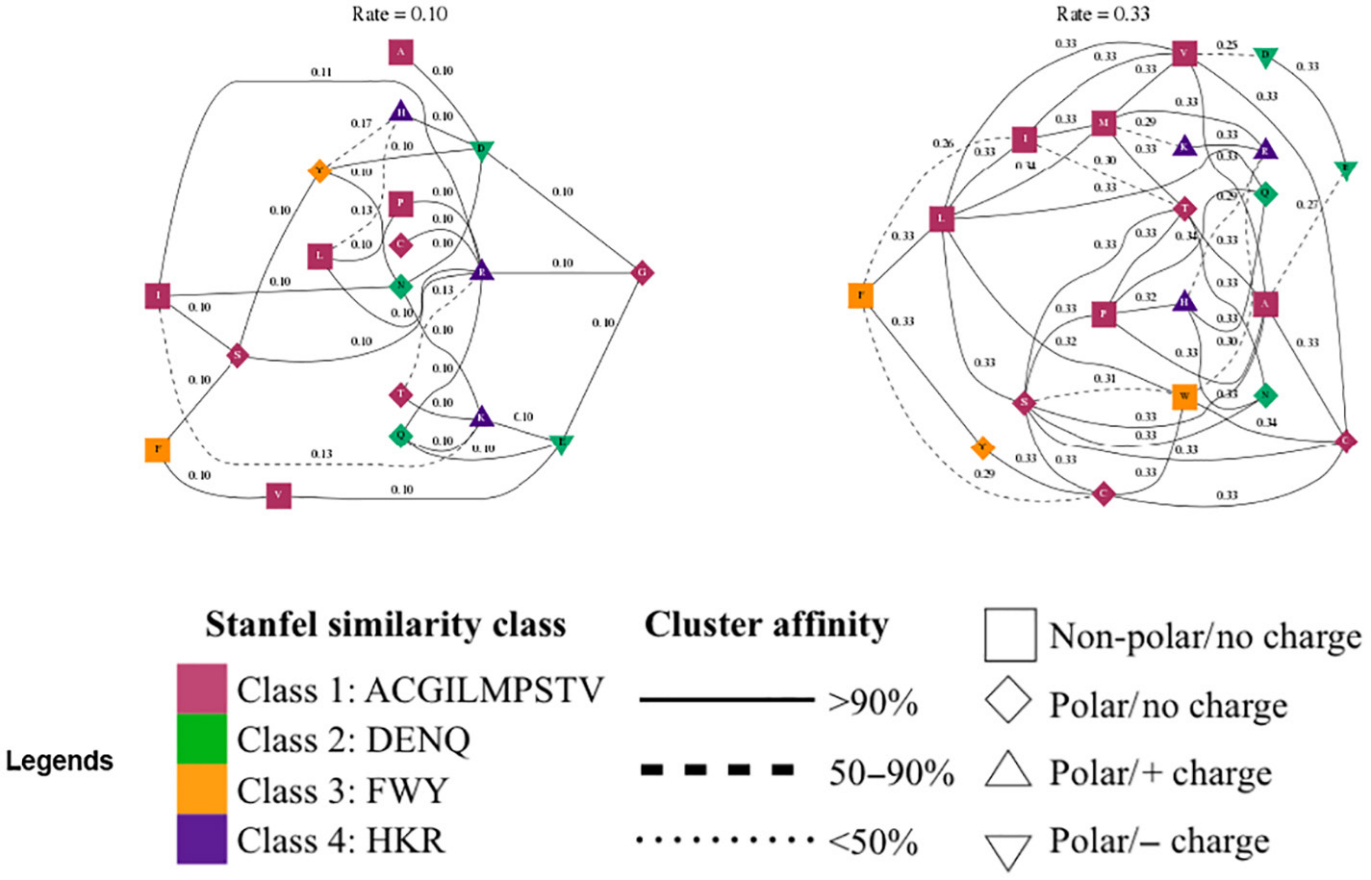
Evolutionary rate clustering in structured genetic algorithm models. The models were inferred from the *PPARGC1A* gene alignment of different species. Each cluster was labeled with the maximum likelihood estimate of its inferred rate. The nodes (residues) are annotated by their biochemical properties and Stanfel class, and the rates (edges) are labeled with the model‐averaged rate estimates.

We further analyzed the Evolutionary rate clustering in structured genetic algorithm models. We found adaptive evolution at the essential amino acid sites in these proteins with various replacement ratios during evolution. The maximum substitution rate for the various ratio groups was 0.33, reflecting the group of amino acids that evolved the fastest within the analyzed proteins. Conversely, the slowest rate of change among the amino acid sites was 0.1. This more conservative rate suggests that certain amino acid sites within the *PPARGC1A* gene are more evolutionarily conserved, likely because changes at these sites could be detrimental to the protein's function (Figure 1).

Overall, the analysis suggests that while there is a range of evolutionary change rates at different sites within the *PPARGC1A* gene's proteins, ultimately, there is evidence of adaptive changes that are presumed to be beneficial and thus selected for throughout evolution.

### 3.7 Phylogenetic analysis

**FIGURE 2:**
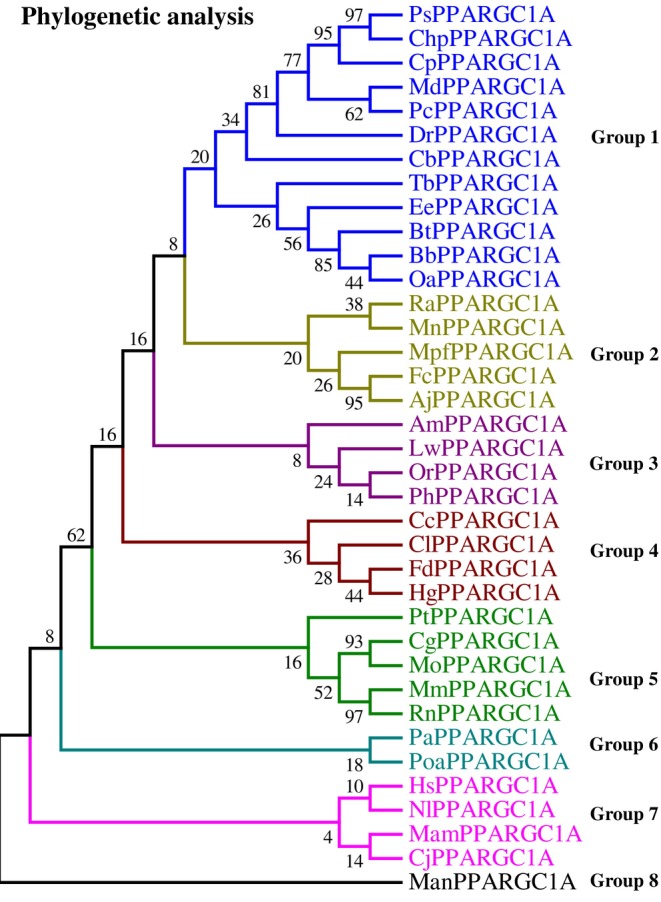
Phylogenetic tree of the *PPARGC1A* gene in different species.

The NCBI database was utilized to download the protein sequences and coding sequences to construct the phylogenetic tree (Figure 2). In the constructed tree, it is indicated that the evolutionally close genes form groups with different species with less significant relationships making up various groups. The figure shows the relationship between the *PPARGC1A* gene of different species. In the resulting phylogenetic tree, eight main clades can be seen. In the first group, 12 genes were studied. In this group, Bootstrap is 97% between ps and chp. In the second group, five genes are observed that Bootstrap is seen above 90% between fc and Aj. In the third group, our genes, in the fourth group, four genes, and in the fifth group high boots between mm and pn are observed. Two and four genes were observed in the sixth and seventh groups, respectively. Bootstrap is also low in these two groups.

### 3.8 Ramachandran

**FIGURE 3:**
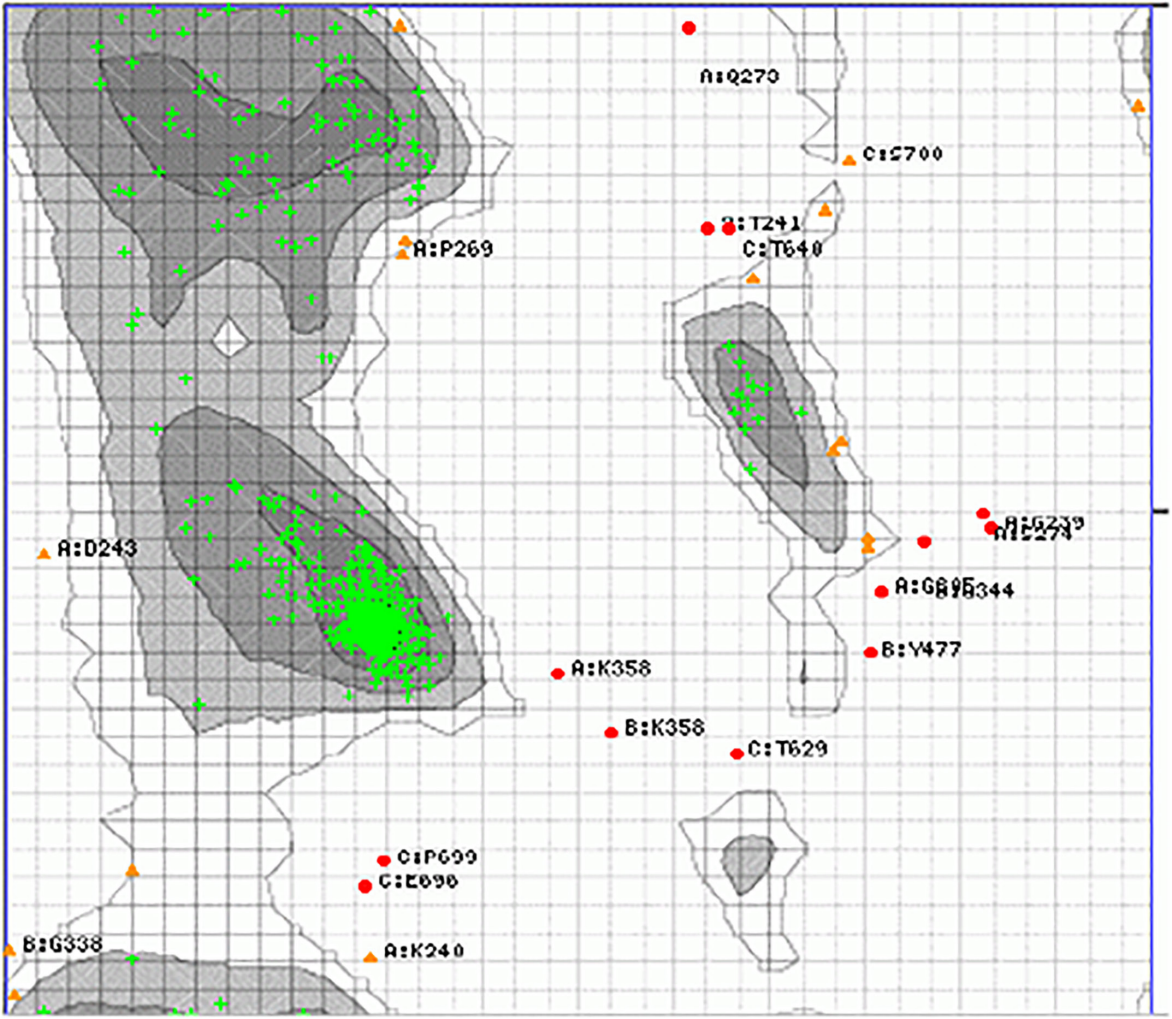
Ramachandran plot predictation to examine the protein structure, the conformation of the amino acid present in the protein, and close associates between the atoms: Black, gray, dark gray, and light gray show highly preferred conformations. Delta > = −2. White with black grid shows preferred conformations. −2 > Delta > = −4. White with gray grid shows questionable conformations. Delta < −4. Highly preferred observations shown as Green crosses: 512 (94.640%). Preferred observations are shown as Brown triangles: 16 (2.957%). Questionable observations shown as Red circles: 13 (2.403%).

We also utilized the Ramachandran map to predict the *PPARGC1A* gene application (Figure 3). A Ramachandran scheme is utilized to visualize energy‐permissible regions for a torsion angle of the *psi* polypeptide (ψ) against the *phi* (φ) amino acid residue present in a protein structure. The main N‐Calpha and Calpha‐C bond chains of the Ramachandran design polypeptide have free rotation. The relative torsion rotation angles were denoted by *phi* and *psi*, respectively. In nature, peptide bonding is rigid and flat. To understand Ramachandran, it is crucial to know the structure of peptide bonding. The analysis of the protein structure the central role of some amino acids, and the close contact of the atoms in the protein are displayed in Figure 3.

### 3.9 Motif analysis

**FIGURE 4:**
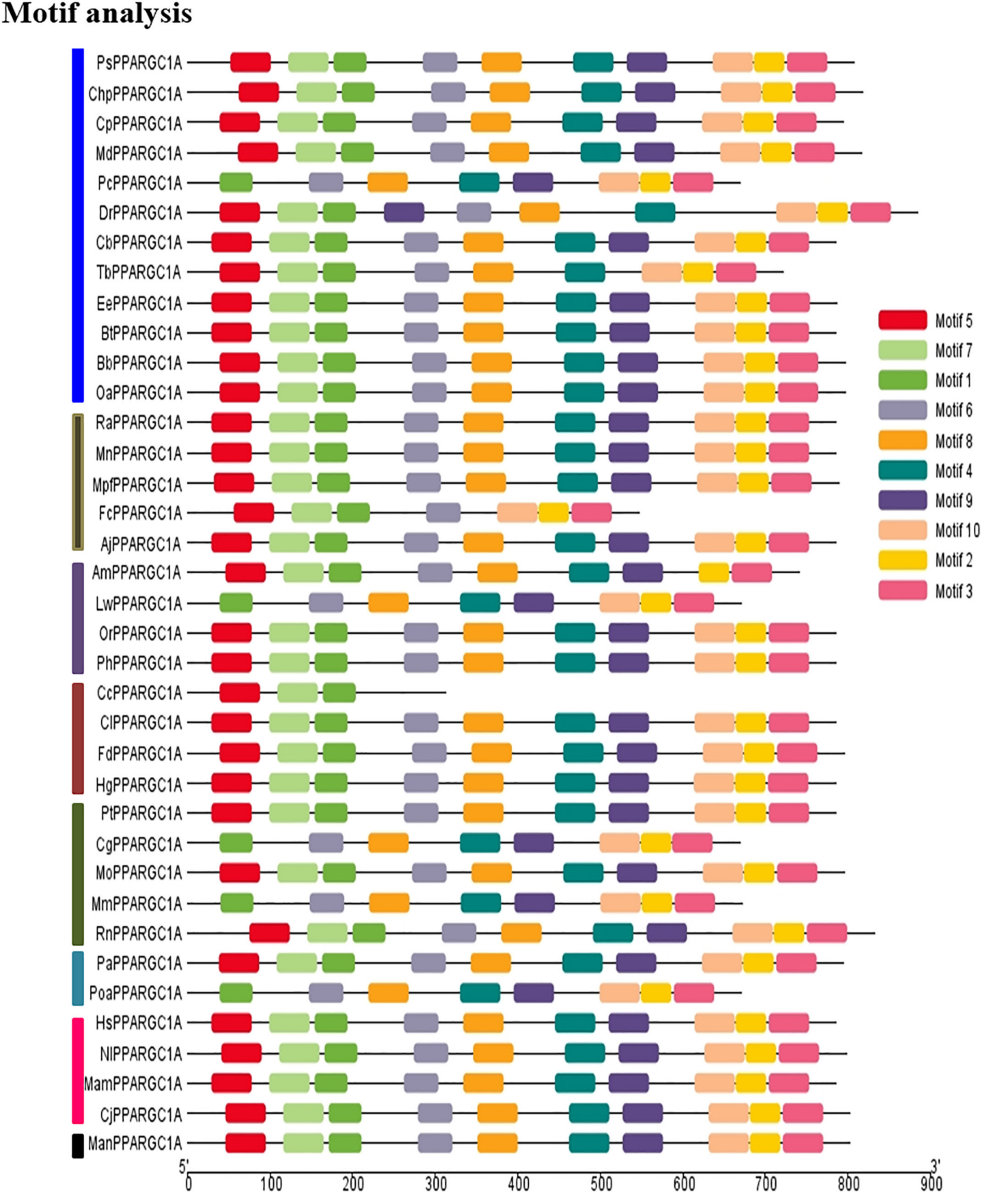
Conserved region analysis in *PPARGC1A* gene from different species.

The results of these analyses are shown in Figure 4. A total of eight motifs were identified. This analysis showed that the pattern of placement of the motifs corresponds to the phylogenetic tree. However, in the first group in the gene related to the pc species, there is a difference in the pattern placement in the third group in lw and the fourth group in cc.

### 3.10 Protein–protein interaction

**FIGURE 5:**
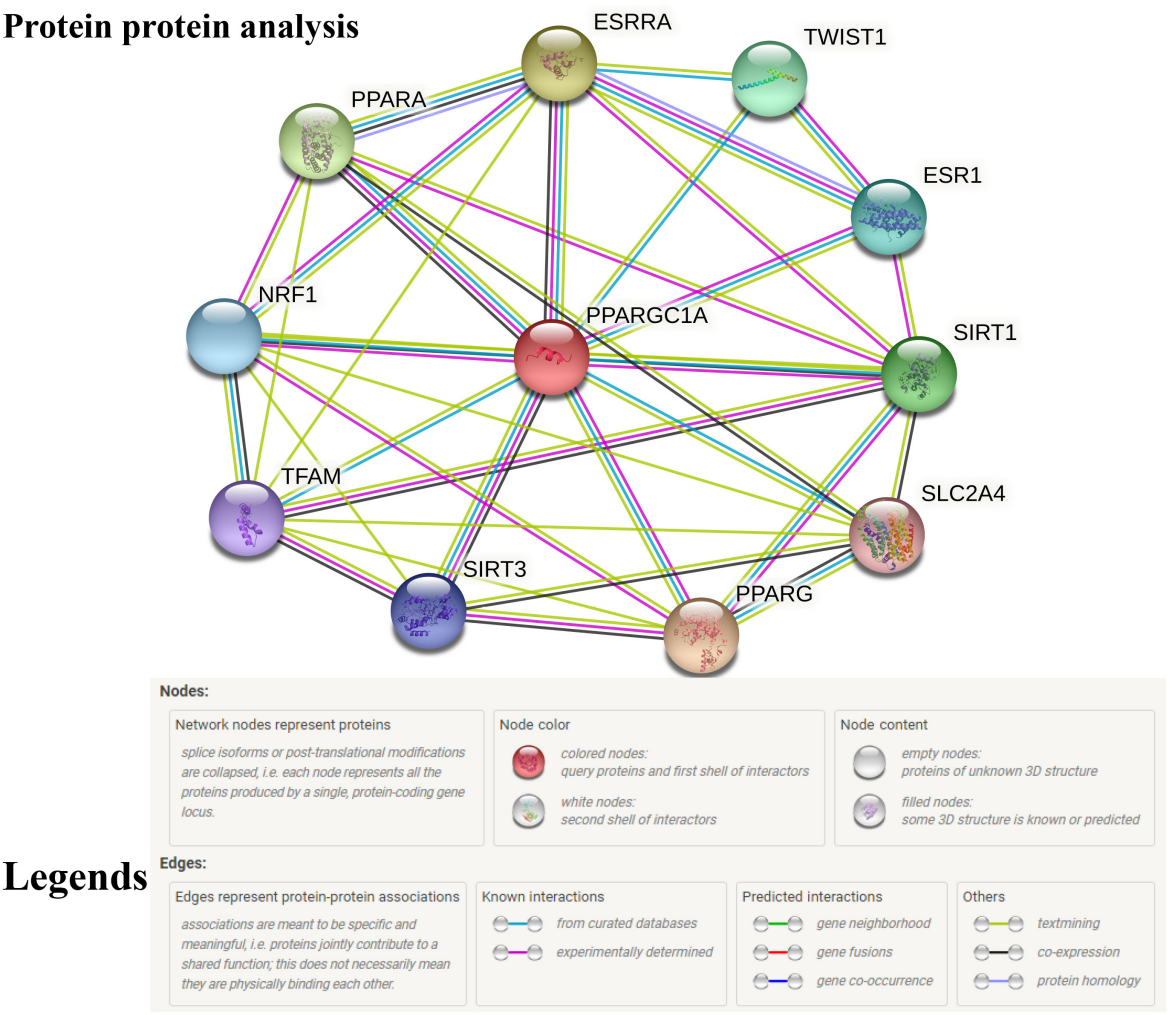
Protein–protein interaction (PPI) network generated by the STRING database for the PPARGC1A gene. Red and gray circles indicate the upregulated and downregulated genes, respectively. Solid edge mean positive and negative correlation coefficient. Line thickness indicates the strength of the interaction. Network nodes denote proteins' post‐transcriptional modifications or splice isoforms, and every node shows all the proteins produced by a single, protein‐coding gene locus.

The results in Figure 5 are from a protein–protein interaction (PPI) network analysis, which gives an insight into the complexity and connectivity of interactions among the proteins in the study.

The network comprised 11 nodes, each representing a single protein, and in some instances, a group of proteins, including those of *PPARGC1A* (peroxisome proliferator‐activated receptor gamma coactivator 1‐alpha), *PPARG* (peroxisome proliferator‐activated receptor‐gamma), *ESRRA* (steroid hormone receptor ERR1), *PPARA* (peroxisome proliferator‐activated receptor alpha), *SIRT1* (NAD‐dependent protein deacetylase sirtuin‐1), *TWIST1* (twist‐related protein 1), ESR1 (estrogen receptor), *NRF1* (nuclear respiratory factor 1), *SIRT3* (NAD‐dependent protein deacetylase sirtuin‐3), *TFAM* (transcription factor A), and *SLC2A4* (Solute carrier family 2). Additionally, we found 36 interactions (edges) between the proteins (nodes). An edge represents a physical or functional association between two proteins. On average, each protein interacts with 6.55 other proteins. The “node degree” is a measure of connectivity, so a higher average node degree suggests that proteins in the network have many interactions with other proteins. Furthermore, the average local clustering coefficient is 0.826 on a scale from 0 to 1, where 1 would indicate that a node's immediate neighbors are all interconnected. A high clustering coefficient suggests that the proteins tend to cluster together, indicating a tightly knit group where each protein is likely to be connected to many others in the group, forming “cliques.” In a random network with the same number of nodes, you would expect there to be 13 interactions. This is significantly fewer than the observed 36, suggesting a non‐random pattern of interactions. Additionally, the PPI enrichment *p*‐value of 1.77e‐07 (.000000177) suggests that the observed number of interactions (edges) between these proteins is extremely unlikely to have happened by chance.

### 3.11 Domain analysis

**FIGURE 6:**
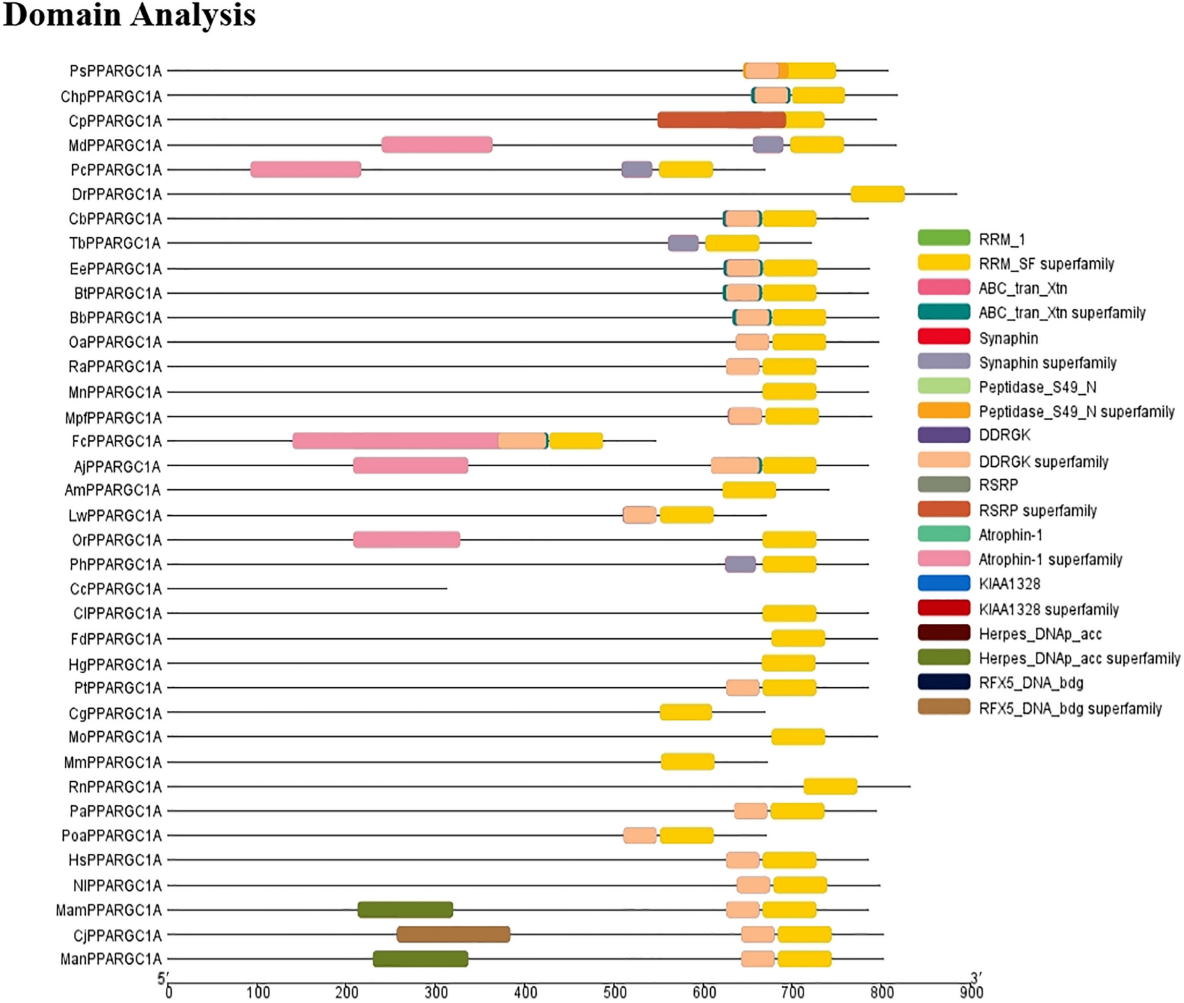
Comparative Domain Analysis of the *PPARGC1A* Gene Across Diverse Speies. The domain structure was constructed through TBTool v1.068 (Chen et al., 2020) to confirm the *PPARGC1A* respective domain in each sequence.

The results of the visualization domain are shown in Figure 6. As can be seen, the RRM‐SF and DDRGK domains are found in almost all species, which shows the similarity of different species in their domains.

Overall, the achievement of methods depends on several factors, including the availability of Candida genes. Evaluate the viability of genetic diversity in them and the number of loci involved in particular trait evolution. The rich selection of the proposed candidate genes appears to be important in terms of their phenotypic effects, and detrimental effects at known sites in same‐sex captive lines may not always be shown in captivity.

### 3.12 Codons cumulative behavior

**FIGURE 7:**
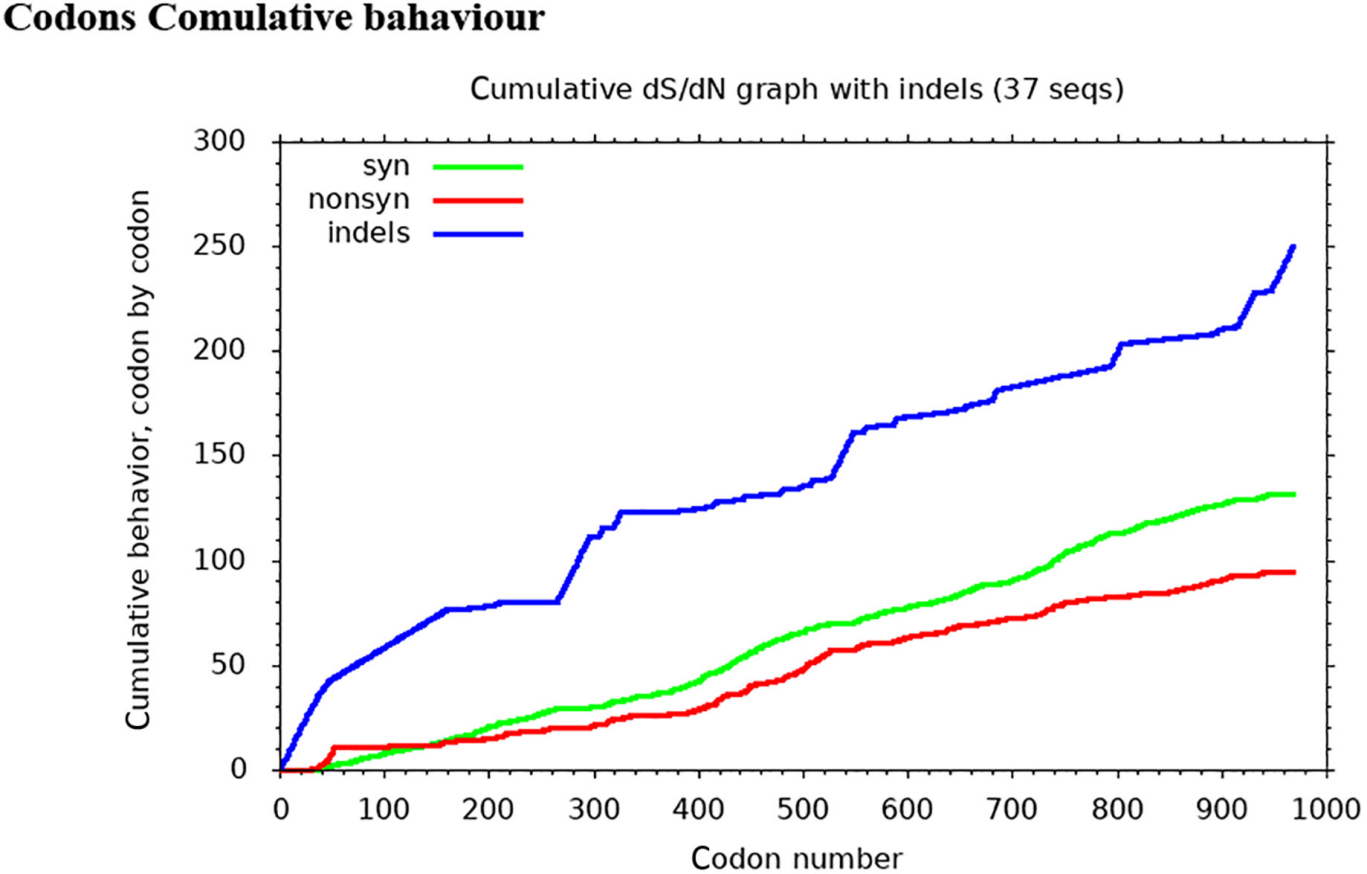
Cumulative behavior of synonymous, nonsynonymous, and ambiguous codon mutation changes per site compared the mutation changes estimates between several synonymous, nonsynonymous mutations and indels (ambiguous codons) per site. The red plot shows the nonsynonymous, green synonymous, and blue for ambiguous codon positions in each group.

The collective function of the identified codon locations is plotted (Figure 7). The collective function of the obscure, synonymous, and nonsynonymous codon deviation with the evolutionary time unit is shown. For the number of start codons, the synonymous mutation's collective performance decreases and then amplifies with the evolution of the codons. In contrast, the unnamed codons' performance increases with the evolutionary unit time according to the position of the codon, but in the position at first and then gradually increases. The performance of ambiguous codons increases with the starting position of the codons and then is fixed.

### 3.13 Selection pressure of *PPARGC1A* gene sequences

**FIGURE 8:**
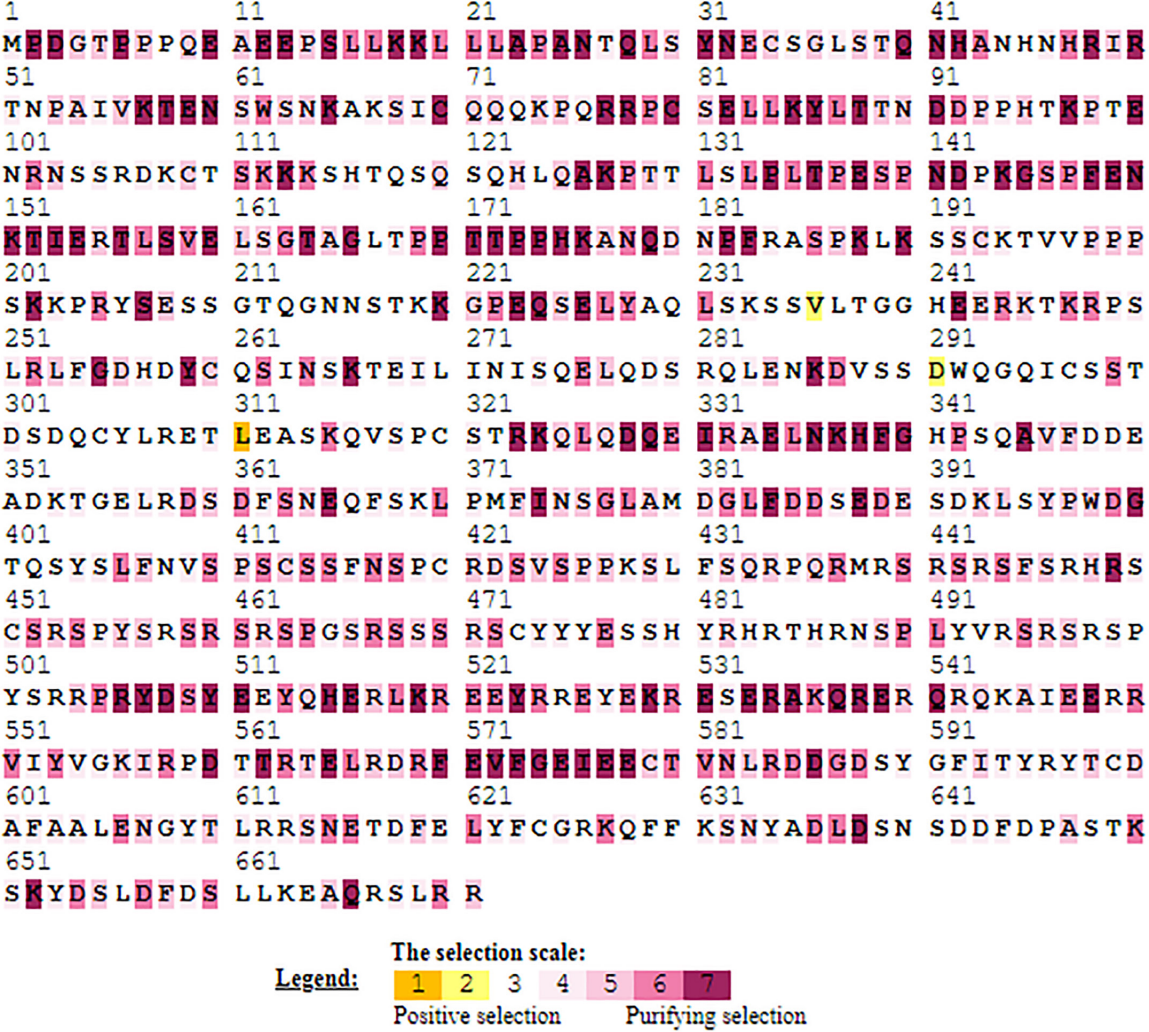
Exploring Evolutionary pressures among goat *PPARGC1A* gene sequences: Insights from Mechanistic‐Empirical Selection Analysis.

The mechanistic‐empirical combination (MEC) model, a selection tool, was utilized to examine the selective pressures acting on the *PPARGC1A* gene sequences. Brown and yellow highlights indicated sites under positive selection, while purple highlights denoted sites under negative or purifying selection. The neutral selection was marked by white and gray highlights. Of the various methods employed in this study, three codon sites within the *PPARGC1A* gene were identified as being under positive selection. The selection indices were characterized by a predominance of conserved amino acids. However, a considerable number of amino acids were found to be under positive selection. The selection outcomes, depicted with color scales, are displayed in Figure 8.

## 4 DISCUSSION

The foundational genetics of various traits in different species have been elucidated through the candidate gene approach. Discovery of such genes plays a crucial role in understanding the phenotypic variations within livestock populations. This not only offers new insights into evolutionary processes but also sheds light on positive selection mechanisms (Brown et al., 2013). Signatures of positive selection within genomic regions that play significant roles can complicate our understanding of genetic variations. Consequently, exploring such regions will offer substantial support for identifying genetic variations, which would facilitate the dissection of these functional regions and advance our knowledge of phenotypic diversity (Ahmad et al., 2017; Brown et al., 2013).

The current study aimed to explore the molecular basis of evolution and the impact of positive selection on the *PPARGC1* *A* gene. This was achieved by analyzing the gene's codon usage and sequence and comparing the nonsynonymous to synonymous substitution ratio using two distinct maximum likelihood methods (Ahmad et al., 2018, 2019; Farmanullah et al., 2020).

In our maximum likelihood analysis, the observed positive dN‐dS values at codons 4, 11, 66, and 123 suggest an evolutionary advantage conferred upon organisms by nonsynonymous mutations at these sites. The uniformity of the dN‐dS values (0.50) across these diverse codons is indicative of a consistent selective advantage. These mutations may plausibly contribute to fitness advantages, potentially enhancing interactions with ligands or altering conformation dynamics, leading to adaptive variations in metabolic pathways. The distinctively high dN‐dS value of 1.96 at codon 137 spotlights this site as a significant evolutionary landmark within the *PPARGC1A* gene. This value suggests an exceedingly strong positive selection that may be pivotal to the function or regulation of the gene (Suzuki & Nei, 2001). One could infer that the amino acid change at this locus is of great adaptive value, possibly influencing phenotypic traits that afford a survival benefit to the species. In contrast, the minimal dN‐dS values observed at codons 30 and 90 points toward a purifying selection, or at best, evolutionary neutrality. The lack of significant positive or negative selection suggests that these codons are functionally constrained. These may represent well‐conserved regions of the *PPARGC1A* gene where any deviation from the status quo could be detrimental, thereby preventing the accumulation of nonsynonymous changes. After assessing the nonsynonymous and synonymous mutations of the *DRB‐1* gene in Eastern woodrats, evidence of positive selection has emerged, indicating that the *DRB‐1* gene is under positive selection (Klein, 1986). Additionally, Asif et al. (2017) demonstrated that the *IL‐33* gene is under positive selection in goats. Similarly, this study provides significant support that the *PPARGC1A* gene under investigation is also subject to positive selection.

The codon model selection results provide significant insights into the evolutionary dynamics of the *PPARGC1A* gene, highlighting the impacts of natural selection on varying rates of synonymous and nonsynonymous substitutions (Farmanullah et al., 2020). In the current study, the Class 1 Model indicates prevalent purifying selection on the *PPARGC1A* gene due to its high mBIC value and lower nonsynonymous to synonymous rate, suggesting a tendency to conserve gene function. The Class 2 Model improves upon Class 1 by revealing variable selective pressures across codons, as shown by a lower mBIC and the introduction of multiple rate classes. This better reflects the gene's evolutionary complexity. The Class 3 Model, with the lowest mBIC but a negative ΔmBIC, suggests an overly complex representation, yet draws attention to particular codons that might be under adaptive evolution, providing a nuanced view of selection on the gene. In the study by Ahmad et al. (2017), three rate classes detected evolutionary fingerprints of the *MC1R* gene using the modified Bayesian information criterion (mBIC) which employed 6680 models to estimate amino acid exchangeability rates. Models that combined empirical codon data with physicochemical parameters, such as transition/transversion rates, exhibited evidence of selective effects and substitution preferences for specific amino acids (Kosiol et al., 2007). The differences in selection pressures captured by the varying models underscore the significance of applying multiple rate models for a deeper understanding of gene evolution. Such insights could potentially contribute to our knowledge of *PPARGC1A* gene functions, and its role in the adaptive significance to the organisms and might direct future research toward understanding the physiological implications of the distribution of selection pressures across its codons. In Table 4, the analysis offers insight into the *PPARGC1A* gene's evolution, highlighting amino acid changes from differing selection pressures, as evidenced by dN/dS ratios. A dN/dS of 0.10 in 30 rate classes indicates strong purifying selection, where most nonsynonymous changes are suppressed to maintain protein integrity. However, there are significant structural and charge variations when changes occur, pointing to selective evolutionary adjustments that influence the protein's form and interactions, albeit with limited polarity changes, reflecting the sensitivity to alterations affecting its interaction with the cellular environment. A slightly higher dN/dS ratio of 0.33 across 45 rate classes signals a less stringent purifying selection, allowing for more amino acid diversity. This may correspond to protein regions more tolerant to changes, as seen by the greater acceptance of polarity changes and lesser charge modifications, indicating that changing the protein's charge is evolutionarily unfavorable. Ahmad et al. (2017) found evidence of molecular evolution in MC1R coding sequences, which was predictable given their association with regions under positive selection in proximate areas. Our results are consistent with those obtained by Cheviron et al. (2006). Axelsson et al. (2005) suggested that the dN/dS ratio could be elevated by gene conversion events involving genes with GC nucleotide pairs, impacting the apparent rate of evolution. Overall, the variation in selection pressure suggests a conservation strategy that upholds structural and functional integrity while permitting certain evolutionary adaptations. To conclude, the codon‐specific approach adopted in the selection of the best codon model delivers refined insights into the evolutionary mechanics within the *PPARGC1A* gene. These results highlight the complexity inherent in the gene's evolution, where some regions of the gene appear to be under stronger purifying selection, likely reflecting the necessity to maintain critical functions, whereas others show a limited scope for variation that might signify places where evolutionary experimentation is tolerable, perhaps even advantageous. These findings firmly anchor the *PPARGC1A* gene within a framework of evolutionary functionalism, affirming its role as a model of evolutionary constraint intertwined with permissible diversification. The same as our study, Asif et al. (2017) found that an evolutionary study of the *IL‐33* gene across 12 mammal species provides evidence of purifying selection.

In the current study, we enhanced our understanding of the *PPARGC1A* gene's adaptation, with four computational methods highlighting codons under positive selection indicative of functional importance across species. IFEL pinpoints eight sites with significant *p*‐values, reflecting recent selection pressures favoring certain mutations. REL expands on this by using Bayes factors to identify a wider set of codons influenced by selection, where a high value indicates strong evidence of selection. Conversely, FUBAR's conservative Bayesian approach provides high confidence in the 12 sites it identifies as undergoing positive selection. SLAC employs a classic frequentist method, finding three sites with statistically significant low *p*‐values, offering a more conservative but stringent approach to detecting positive selection. Each method contributes to a multifaceted view of the gene's evolutionary dynamics, highlighting areas of adaptability and function. It is compelling to note that site 52 is identified by both IFEL and FUBAR methods, while site 394 is identified consistently across IFEL, FUBAR, and SLAC, which strongly suggests that these sites may indeed experience positive selection. It provides a cross‐validation of the positive selection signals across methods with different assumptions and sensitivity. However, the identification of site 29 solely by REL with strong support and by FUBAR with less certainty —but not by IFEL and SLAC—highlights the complexity of evolutionary inference and the possibility that different statistical tests might capture different facets of selection pressures, or they may be variably affected by the evolutionary history and sequence data quality. Farmanullah et al. (2020) identified the number of positive selection sites for *AKT3* as 33, 418, and 1, using REL, FUBAR, and IFEL, respectively. Ahmad et al. (2017) revealed that REL detected five codons under positive selection at sites 59, 218, 248, 327, and 364, while IFEL identified three codons at sites 59, 279, and 327 associated with the *MC1R* gene. Sites 59 and 327 were detected as positively selected by both methods, whereas sites 218, 248, and 364 were detected only by REL. This discrepancy may occur because other approaches lack the power to analyze datasets containing sequences with low divergence (Pond & Frost, 2005). In another study, SLAC found no sites under selection, while FEL and REL detected two sites under positive selection, indicating fewer false‐positive results (Sorhannus & Pond, 2006). The discrepancies observed between methods underscore the complexity of interpreting adaptive evolution. The reliance on statistical thresholds (such as *p*‐values and Bayes Factors) and different modeling assumptions can affect the identification of selected sites. For example, *p*‐value‐based methods might fail to detect sites under weak positive selection, while Bayesian approaches could detect them based on the strength of the posterior probability.

We used MEME to analyze the *PPARGC1A* gene, offering a granular perspective on how selective forces have acted episodically on specific codons. The concept of episodic diversifying selection is crucial; it suggests that these sites have experienced moments of positive selection, potentially reflecting adaptation to transient environmental pressures or population‐specific advantages, while predominantly being under purifying or neutral selection (Murrell et al., 2012). At codon 33, the synonymous rate is equal to the nonsynonymous rate under purifying selection—both with a relatively high rate of 0.64. The high probability (Pr[*β* = *β*−] = .91) suggests that most of the evolutionary history for this site is characterized by purifying selection, maintaining genetic homogeneity. However, the presence of a much higher nonsynonymous rate possibility (*β*+ = 920.26) with a lower probability (.09) speaks to rare but significant instances of positive selection. The *p*‐value of .00 indicates the deviation from neutrality is highly significant, whereas the *q*‐value of 0.01 underscores the result's reliability against the backdrop of multiple tests. Conversely, codon 394 presents a compelling case, where the probability of positive selection (Pr[*β* = *β*+] = 1.00) is certain, yet the observed positive selection rate (*β*+ = 1.61) is relatively moderate. The *p*‐value and *q*‐value at this site, while still indicating statistical significance, suggest a weaker inference of positive selection compared to codon 33. This indicates that while positive selection is thought to affect codon 394, the evidence is not as compelling when factoring in multiple comparisons, which increases the stringency of the test. Farmanullah et al. (2020) identified 20 sites under positive selection in AKT3 using MEME. In a separate study, Auclair et al. (2013) detected evidence of positive selection of the human BMP15 gene across 24 mammalian species. They further reported that BMP15 evolved rapidly, permitting positive selection at a faster rate among TGF family members within the mammalian clade. Our results reflect the episodic nature of selective pressures on the PPARGC1A gene, pinpointing sites that may have been key players in adaptive evolution. Codons identified consistently with both low *p*‐values and *q*‐values emerge as the most compelling targets for positive selection, hinting at their possible roles in important physiological or developmental adaptations. These findings can inform more focused evolutionary or functional studies to decipher the specific contributions of these sites to the organism's fitness and survival.

Our analysis of the evolutionary rate clustering in structured genetic algorithm models provides a pivotal glimpse into how the PPARGC1A gene has been shaped by evolutionary forces. The detection of adaptive evolution at essential amino acid sites reflects the influence of functional constraints and potential benefits conferred by certain mutations (Delport et al., 2010). The finding that the maximum substitution rate among the various ratio groups was 0.33 indicates that some amino acid sites have undergone rapid evolution. This rate implies that the gene has regions that are relatively less constrained and may tolerate—or perhaps require—faster rates of amino acid replacement to adapt to environmental pressures or new functional demands. Sites with such elevated substitution rates could be important for the gene's ability to permit variation and could be tied to specific adaptations that have provided selective advantages. Conversely, the observation of a more conservative substitution rate of 0.1 at certain sites within these proteins signals strong evolutionary conservation. It suggests that these sites are likely critical to the protein's structure and function. Changes at these highly conserved sites may be more likely to be deleterious, impairing the protein's function or stability, and thus are selected against. This points to the presence of core functional domains within the PPARGC1A protein that have remained relatively unchanged throughout evolutionary history, likely due to their essential roles. In one study, Delport et al. (2010) discovered startling heterogeneity in the substitution rates within the HIV‐1 pol gene: a single rate dN/dS estimate of 0.15 expands into seven rate classes, with relative nonsynonymous substitution rates varying from 0.047 (involving 20 residue pairs) to 1.561 (involving three residue pairs). This range is similarly revealed in other datasets. Overall, our analysis of evolutionary rate clustering underscores the nuanced interplay between conservation and innovation in the genetic evolution of the PPARGC1A gene. Recognizing the presence of both conserved and rapidly evolving sites within the gene provides valuable insights into its functional versatility and adaptability. It also points to potential areas of interest for further research into the gene's role in the organism's physiology, adaptations, and the potential functional consequences of specific amino acid changes in evolutionary history.

The construction of a phylogenetic tree using protein sequences and coding sequences from the NCBI database provides a visual representation of evolutionary relationships between the PPARGC1A gene from various species (Dylus et al., 2022). In the current study, the phylogenetic tree displays eight main clades, indicating distinct evolutionary trajectories for the involved species groups. High bootstrap values across various clades suggest reliable ancestral linkages among genes. Specifically, a 97% bootstrap value for the first group supports a close relation between the ps and chp genes. Similarly, high bootstrap scores for the second group denote a shared evolutionary history among five genes. While high bootstrap values in the fifth group support a robust evolutionary association, lower values in the sixth and seventh groups suggest less certainty in these clade connections. Overall, the clades and bootstrap confidence levels provide insight into the relationships and evolutionary paths of species with the *PPARGC1A* gene. In the study by Asif et al. (2017), the construction of a phylogenetic tree revealed that goat IL‐33 closely resembles sheep IL‐33. Farmanullah et al. (2020) identified codon sites under positive selection within mammalian clades through the study of the phylogenetic tree. She (2011) reported on the study of gene evolution, phylogenetic branch length, and positive selection analysis of *AKT3*. The study utilized the 39 nucleotide coding sequences from various mammalian species to investigate the selection pressure experienced. The analysis that led to the detection of eight distinct motifs within the *PPARGC1A* gene product is an investigation into the conservation and variability of certain functional elements among proteins (Ahmad et al., 2018). In the context of our analysis, the correspondence between the pattern of motif placement and the phylogenetic tree provides evidence that certain motifs have been conserved throughout evolution. It suggests that the proteins' structure and function have been retained within the clades represented in the tree, where closely related species (as inferred from the tree) share similar motif patterns. This is what we would typically expect as species that are closely related are likely to have inherited the same genetic and proteomic features from common ancestors. However, the deviations in the motif placement pattern observed in the first group related to the pc species, as well as the differences noted in the third group in lw and the fourth group in cc, are particularly noteworthy. Such discrepancies could indicate several evolutionary phenomena: Divergent evolution, convergent evolution or parallel evolution, structural or functional specialization, mutation, and genetic drift.

LeMoine et al. (2010) investigated the evolutionary trajectories of *PGC‐1α* homologs in fish and mammals to evaluate this coactivator's evolution. They suggested that the *PGC‐1* family diversified early in vertebrate evolution through repeated genome duplication events, as indicated by a phylogeny of the *PGC‐1* paralogs. Bayesian and maximum likelihood phylogenetic reconstructions of *PGC‐1α* across representative vertebrate species revealed divergent evolutionary dynamics within the protein's different functional domains.

The results from the protein–protein interaction (PPI) network analysis offer valuable insights into the functional landscape of the proteins in our study, particularly in understanding the role of *PPARGC1A* within a complex web of interactions. An 11‐node network with such a high number of interactions (36 edges) suggests that PPARGC1A and the associated proteins (such as *PPARG*, *ESRRA*, *PPARA*, *SIRT1*, *TWIST1*, *ESR1*, *NRF1*, *SIRT3*, *TFAM*, and *SLC2A4*) form a highly interconnected system. Such a high‐density network reflects the central involvement of these proteins in various cellular pathways and potentially their collective importance to the regulation of cellular metabolism, gene expression, and other pivotal biological processes. An average node degree of 6.55 indicates that each protein is likely to be involved in multiple interactions. High connectivity points to a certain robustness of the network, with multiple pathways potentially compensating for a loss or gain of function in one protein. This redundancy can be critical to maintaining cellular functions despite various stresses or mutations. The high average local clustering coefficient of 0.826 highlights that the network is far from random. It suggests that proteins do not interact haphazardly but rather form specific groups or modules within the network. Such a modular network might imply that proteins within a cluster work together in specific biological functions or processes. For instance, proteins in a cluster could be part of a signal transduction pathway or a metabolic network, where each member's role is coordinated with the others. In a comparable random network, the expected average number of interactions would be notably lower (13 interactions), which starkly contrasts with the 36 interactions observed in our study. This underpins the argument that the network's proteins are not interacting randomly but likely have evolved to interact due to a shared functional context (Ahmad et al., 2019). The extremely low PPI enrichment *p*‐value of 1.77e‐07 reaffirms that these interactions are not a product of random chance. This statistical significance implies that the network's dense interconnectivity is a result of biological necessity, likely reflecting evolutionary pressures that favor co‐regulated or functionally synergistic interactions (Farmanullah et al., 2020). The implications for *PPARGC1A*'s role in the network are significant. Given the interconnected nature of these proteins, *PPARGC1A* is likely integral to the co‐regulatory processes governing energy metabolism, mitochondrial biogenesis, and other related functions governed by this network of proteins. It could stand as a potential pharmacological target for diseases linked to these pathways due to its pivotal role within the network. Such a PPI network analysis can be a springboard for further investigations. For example, in diseases where the *PPARGC1A* pathway is disrupted, understanding the network's connectivity may help in designing interventions that target not only *PPARGC1A* but also other proteins within the network to restore normal function or compensate for the dysfunction. However, many genes identified as potentially under positive selection have been implicated in the cancer process (Nielsen et al., 2005). Genes associated with complex diseases, such as asthma‐interleukin‐13 and type 2 diabetes (*CAPN10*) (Fullerton et al., 2002), have also been reported to exhibit signatures of positive selection in various studies. Gaur et al. (2017) performed a 3D structural analysis of the PARP1 and PARP2 proteins, revealing that certain positively selected sites altered the proteins' electrostatic potential. Such changes may affect their interactions with other proteins and molecules, potentially leading to functional differences. Gaur et al. (2017) investigated the molecular evolutionary patterns of selection signatures in 51 species for 10 genes significant to NAD+/Sirtuin pathways in worms and mice. They discovered that *MRPS5* and *PPARGC1A* were subject to significant constraints owing to their functional importance. Overall, these findings powerfully illustrate the interconnectedness of cellular pathways and offer a map for unraveling the molecular mechanisms by which PPARGC1A and its associated proteins execute their biological roles.

The visualization of domain conservation across different species in our study, revealing the presence of RRM‐SF (RNA recognition motif superfamily) and DDRGK (DDRGK domain‐containing protein) domains in almost all species studied, is a significant observation. It indicates that these domains are highly conserved across various species, suggesting they play fundamental roles in cellular processes that are vital across a broad range of organisms (Lorković & Barta, 2002). The RRM‐SF is known for its involvement in post‐transcriptional gene expression processes, including RNA binding, splicing, and stability, which are critical functions conserved across eukaryotes. The DDRGK domain's function is less well‐characterized but is often involved in protein–protein interactions and may play a role in ubiquitination processes. The conservation of these domains underscores their potential importance in maintaining essential biological functions. The MEC model analysis of the *PPARGC1A* gene in our study reveals three codons under positive selection, suggesting evolutionary adaptation possibly linked to environmental challenges influencing organism fitness. Conserved amino acids, subject to purifying selection, reflect the gene's critical role in essential functions like energy metabolism. Meanwhile, positive selection in certain regions indicates evolutionary flexibility amidst primarily conserved protein functions. Neutral regions appear to be evolving without currently impacting fitness, potentially acquiring selective significance in the future. This MEC model analysis gives insights into the adaptive evolution of PPARGC1A and informs potential areas for functional research. Farmanullah et al. (2020) measured the adaptive selection pressure at codons in the *AKT3* sequence and employed the mechanistic‐empirical combination (MEC) model to identify codons under positive selection.

LeMoine et al. (2010) suggested that the PGC‐1α protein in vertebrates has undergone modular evolution, potentially permitting lineage‐specific divergences in the coactivating capabilities of this regulator.

Understanding the nuances of how the PPARGC1A gene is evolving helps predict the genetic basis of related phenotypes and may impact the discovery of therapeutic targets or the breeding of species with desirable traits. It also adds to the field of precision medicine, where such data could inform patient‐specific treatment based on their genetic makeup and the variants of this gene they possess.

## 5 CONCLUSION

In conclusion, this study has shed light on the adaptive changes within the *PPARGC1A* gene, pointed to the conservation of essential domains, and revealed a high degree of evolutionary rate variability and interaction complexity. It has also uncovered the gene's pivotal regulatory role, possibly implicating it in various biological processes. The evolutionary significance of this gene is evidenced not only by direct signs of selective advantage but also by integrative analyses that encompass phylogenetics, system biology, structural biology, and evolutionary modeling. Looking forward, these insights into the *PPARGC1A* gene could serve as signposts for further investigation into ecological adaptations, species survival strategies, and the intricate evolutionary pathways that shape life.

## AUTHOR CONTRIBUTIONS

**Seyed Mahdi Hosseini:** Investigation (equal); methodology (equal); visualization (equal); writing – original draft (equal); writing – review and editing (equal). **Farman Ullah:** Formal analysis (equal). **Muhammad Zulfiqar Ahmad:** Methodology (equal). **Majid Pasandideh:** Validation (equal). **Aixin Liang:** Data curation (equal). **Guohua Hua:** Project administration (equal). **Liguo Yang:** Funding acquisition (equal); supervision (supporting).

## FUNDING INFORMATION

This study was funded by the National Key R & D Program of China (2022YFD1301001).

## CONFLICT OF INTEREST STATEMENT

All authors do not have any potential conflict of interest related to this research work.

## Data Availability

The data used to support the findings of this study are included in the article.
